# The role of urine pregnancy testing in facilitating access to antenatal care and abortion services in South Africa: a cross-sectional study

**DOI:** 10.1186/1471-2393-6-26

**Published:** 2006-08-07

**Authors:** Chelsea Morroni, Jennifer Moodley

**Affiliations:** 1 Women's Health Research Unit, School of Public Health and Family Medicine, University of Cape Town, 7925, Cape Town, South Africa

## Abstract

**Background:**

Effective confirmation of pregnancy is a basic component of reproductive health services. It is a prerequisite for accessing antenatal care (AnC) if the pregnancy is wanted and abortion services if the pregnancy is unwanted. This study examined the role of urine pregnancy testing in the timing of presentation for pregnancy-care.

**Method:**

A cross-sectional study was conducted among 158 women presenting for antenatal care and 164 women presenting for abortion at public sector clinics in Cape Town, South Africa.

**Results:**

The median gestational age at first presentation was 23 weeks for AnC clients and 13 weeks for abortion clients. Obtaining a urine pregnancy test of one's own accord was associated with a decrease in the gestational age at presentation of 3.6 and 1.4 weeks for antenatal and abortion clients, respectively, independently of all other factors.

**Conclusion:**

Given the proven clinical benefit and public health impact of early presentation for antenatal and abortion services, strategies to decrease gestational age at presentation for pregnancy care should be given priority. 'Fast-track' urine pregnancy testing services should be established in public sector clinics in South Africa.

## Background

Effective confirmation of pregnancy is a basic component of reproductive health services [1-3]. It is a prerequisite for accessing antenatal care (AnC) if the pregnancy is wanted and abortion services if the pregnancy is unwanted. Pregnancy testing is also often needed to rule out pregnancy prior to contraception [4]. While blood pregnancy tests or clinical examination may be used, administration of sensitive beta-human chorionic gonadotrophin (beta-HCG) urine pregnancy tests is an effective and low-cost means of early pregnancy confirmation that is feasible in many resource-limited settings [4,5].

It is widely believed that urine pregnancy testing, though theoretically available at all public health facilities in South Africa, is under-utilized by clinic staff. Lack of early pregnancy confirmation services may contribute to late antenatal care and late abortion presentations, which are the norm in South Africa and many other parts of sub-Saharan Africa [2,6].

Despite the importance of early pregnancy detection, little is known about how the availability and accessibility of early pregnancy confirmation services, in the form of urine pregnancy testing, may affect gestational age at presentation for AnC and abortion services. We examined the role of urine pregnancy testing in the timing of presentation for public sector AnC and abortion services in South Africa.

## Methods

The study was conducted in two health sub-districts in the greater Cape Town area of South Africa. Public health facilities in these sub-districts include: 18 primary care facilities (where women should be able to obtain a urine pregnancy test) and three maternity units that provide AnC services. These primary level, nurse-run maternity units provide all public-sector AnC care and delivery services for 'low-risk' pregnancies in the sub-districts. All pregnant women obtaining care in the public sector, regardless of 'risk' make their initial pregnancy-related visits to such a facility. First trimester abortion services are provided at two of the primary care facilities (nurse-run clinics that provide all basic primary care services) and second trimester abortion services are provided at a secondary-level referral hospital that serves both sub-districts. Services at all of these facilities are provided free of charge.

A cross-sectional study was undertaken in two groups of pregnant women attending six of the public clinics in the two urban areas: women making their first visit for either 1) AnC services or 2) first trimester or second trimester abortion services.

Over a three-week period, consecutive consenting women, who were age 14 years or older, at all of the facilities providing these services in the study areas were interviewed and asked questions on timing, method and location of pregnancy confirmation, and on referral pathways to current point of service. Refusals were minimal (<3%). Participants were interviewed in their home language using a standardized, pre-tested questionnaire. As is the practice in public clinics in this setting, among AnC attendees, gestational age was dated using information from physical examination and history, whereas gestational age for abortion clients was assessed by ultrasound.

In addition at eight randomly selected public clinics in the two areas, we conducted in-person interviews with healthcare providers using a structured instrument. We also conducted a telephonic survey among providers in a random sample of 50% of public clinics the greater Cape Town area. In total 78 providers, (72 professional nurses, 3 enrolled nurses and 3 health promotion officers) were interviewed. The provider surveys collected basic information about the availability and provision of pregnancy testing services at the clinics, as well as unprompted information about providers' opinions of the advantages and disadvantages of offering urine pregnancy testing in public sector clinics.

Sample size calculations were conducted for one outcome: the change in gestational age at first visit associated with obtaining a urine pregnancy test. The calculation was based on the following assumptions: 1) an average gestational age at first visit among all women of 24 weeks for AnC clients and 12 weeks for abortion clients, 2) a minimum clinically relevant change in gestational age at first visit due to obtaining a urine pregnancy test of two weeks, 3) at least one-third of subjects having obtained a urine pregnancy test, and 4) a two-sided alpha of 0.05, with 80% power to detect the difference. Under these assumptions, 81 women attending for AnC and 81 women attending for abortion were required. This sample size was exceeded in both groups.

Data analysis was conducted using the statistical programme Stata 9.0 (Stata Corporation, College Station, Texas). Bivariate analyses employed *Wilcoxon rank sum *and *χ*-square tests. Separate multiple linear regression models were developed to examine how demographic, behavioural, and health service-related factors were associated with gestational age at presentation for AnC and abortion services.

All participants provided written informed consent, and approval to conduct the survey was granted by the Research Ethics Committee of the University of Cape Town.

## Results

### Socio-demographic characteristics

A total of 322 women participated in the study: 158 at first AnC visit. Eighty-two presenting for first trimester and 82 presenting for second trimester abortion. The participants were young women (median age 24 years; range 14–43). Twenty percent of AnC clients (n = 32), 20% of first trimester abortion clients (n = 16) and 24% of second trimester abortion clients (n = 20) were teenagers. Most participants had had previous pregnancies (n = 203, 63%), were currently in a relationship (n = 248, 77%) and spoke Xhosa (n = 193, 60%) or Afrikaans (n = 90, 28%) as their main language. Only six women reported having ever had an abortion. Women seeking abortion were more likely to be unmarried (63% vs. 91%, p < 0.001), employed (50% vs. 31%, p = 0.001) and to speak Xhosa (68% vs. 52%, p < 0.01) than women presenting for AnC.

### Knowledge of pregnancy testing and timing of presentation for care

Overall, about half of women spontaneously mentioned urine pregnancy testing as a way for a woman to detect a pregnancy, but almost all had heard of urine pregnancy testing when asked directly (Table 1). However, most women did not know that a urine pregnancy test could detect a pregnancy at about two weeks after a missed period. About half of women thought that a urine pregnancy test could only be used to detect pregnancy greater than two months after a missed period.

**Table 1:** Knowledge and practice of urine pregnancy testing among public sector ANC and abortion clients in Cape Town, South Africa

*Characteristic*	*ANC n = 158 (%)*	*Abortion clients n = 164 (%)*
Spontaneously mentioned urine pregnancy testing as way for a woman to detect her pregnancy/know she might be pregnant	79 (50)	88 (54)
Heard of urine pregnancy testing when asked directly	148 (94)	154 (94)
How long after missed period before a urine pregnancy test can detect pregnancy		
1–2 weeks	17 (11)	36 (22)
1 month	55 (35)	60 (37)
2 months	49 (31)	32 (20)
3 months	17 (11)	6 (4)
Don't know	20 (12)	30 (18)
Willing to pay for urine pregnancy test at public clinic	108 (68)	117 (71)
Appropriate time after fall pregnant to make first visit for pregnancy care		
Immediately/as soon detect pregnancy	18 (12)	27 (17)
≥ 1 month	8 (5)	17 (10)
≥ 2 months	12 (8)	41 (25)
≥ 3 months	39 (24)	52 (32)
≥ 4 months	25 (15)	18 (11)
≥ 5 months	23 (15)	9 (6)
≥ 6 months	33 (21)	0
Urine pregnancy test from pharmacy in this pregnancy	42 (27)	63 (38)
Sent from a public sector clinic to pharmacy to obtain pregnancy test and then return to clinic with result	21 (13)	59 (36)

Women's knowledge of the appropriate time in pregnancy to make the first health service visit was low: 86% (n = 136) in the AnC sample and 48% (n = 79) in the abortion sample thought the first visit should be made at three months of pregnancy or later (Table 1).

### Characteristics of current pregnancy

Median gestational age at first presentation was 23 weeks (inter-quartile range (IQR) 18–27) for AnC clients. Only 9 AnC clients (5.8%) presented in the first trimester. Median gestational age at presentation for abortion clients was 13 weeks (IQR 9–15).

For the majority of participants (AnC: n = 87, 55%, abortion: n = 94, 57%) confirmation of pregnancy and first pregnancy-related healthcare visit occurred outside the public health services. AnC clients attended a median of 2 other health facilities before presenting at the current public clinic to initiate pregnancy care, while abortion clients attended a median of 2.5 other facilities. Twenty-seven percent (n = 43), and 38% (n = 63) of AnC and abortion clients, respectively, on their own, obtained a urine pregnancy test at a private pharmacy during this pregnancy. Additionally, many participants reported that during this pregnancy, they had been sent from a public clinic to purchase a pregnancy test at a pharmacy and then return to the public clinic with the results before care would be initiated (AnC clients: n = 21, 13%, abortion: n = 59, 36%). Importantly in all of the clinics where women reported that this practice took place providers reported that pregnancy tests were available (data not shown).

### Pregnancy testing and gestational age at presentation

In bivariate analysis, obtaining a urine pregnancy test of one's own accord at a private pharmacy was associated with a 5-week and 1.5-week decrease in gestational age at presentation for AnC and abortion clients, respectively. On the other hand, being sent from a public clinic to have a pregnancy test at a private pharmacy and return with the results was associated with a 3-week increase in gestational age at presentation among AnC clients and a 1.5-week increase in gestational age at presentation among abortion clients.

In multivariate analysis, several factors were shown to be independently associated with gestational age at first presentation to a public clinic for pregnancy-care, and these factors were similar across the three samples of pregnant women (Table 2). Obtaining a urine pregnancy test of one's own accord from a private pharmacy was associated with a 3.6-week decrease in the gestational age at AnC presentation and a 1.4-week decrease in gestational age at presentation for an abortion. Being sent from any clinic to obtain a pregnancy test at a private pharmacy and return with the results increased gestational age at presentation by 2.8 weeks among AnC clients and 1.2 weeks among abortion clients. Women older than 20 years booked significantly earlier for both AnC and abortion services than teenagers, and for both AnC and abortion services isiXhosa speakers booked significantly later than women speaking either Afrikaans or English.

**Table 2:** Independent predictors from multiple linear regression models of gestational age at booking among public sector ANC and abortion clients in Cape Town, South Africa

ANC clients* Characteristic	*Decrease in number of weeks gestational age at booking for ANC*	*95% CI*	*p-value*
Obtaining a urine pregnancy test at private pharmacy			
No	(Reference category)		
Yes	-3.6 weeks	-5.2; -0.7	0.01
Age			
<20	(Ref)		
20+	-2.7 weeks	-5.3; 0.0	0.05
Main language spoken			
isiXhosa	(Ref)		
Afrikaans	-3.5 weeks	-5.8; -1.2	<0.01
English	-5.4 weeks	-9.1; -1.2	<0.01
Sent from public clinic to obtain pregnancy test at private pharmacy			
Yes	(Ref)		
No	-2.8	-5.4; 0.0	0.05

TOP clients** Characteristic	*Decrease in number of weeks gestational age at booking for abortion*	*95% CI*	*p-value*

Obtaining a urine pregnancy test at pharmacy			
No	(Ref)		
Yes	-1.4 weeks	-2.6; -0.1	0.03
Number of previous pregnancies			
0	(Ref)		
1+	-0.5 weeks	-1.0; -0.4	0.04
Main language spoken			
isiXhosa	(Ref)		
Afrikaans	-1.5 weeks	-2.9; -0.04	0.04
English	+0.7 weeks	-1.2; 2.6	0.5
Other	-2.4 weeks	-6.0; 1.2	0.2
Sent from public clinic to obtain pregnancy test at private pharmacy			
Yes	(Ref)		
No	-1.2	-2.4; -0.2	0.03

*Adjusted for language, level of education, employment status, source of financial support, prior pregnancy, and knowledge of other reproductive health services.

**Adjusted for age, level of education, employment status, source of financial support, employment status, and knowledge of other reproductive health services.

### Provider knowledge and attitudes regarding urine pregnancy testing services

The majority of the 78 public healthcare providers interviewed (n = 69, 89%) reported that pregnancy tests were available at their clinics, however less than half of the providers where pregnancy testing is available (n = 33, 48%) said that there were written pregnancy testing guidelines for their clinics. Less than half of providers had ever been trained on when (n = 37, 48%) or how (n = 31, 40%) to do a urine pregnancy test. Only 53% (n = 41) thought that they should always give a pregnancy test if a client requested one. Fifty percent of providers (n = 39) thought they should not do tests on young teenagers because "they should not be sexually active or using contraception anyway". Most providers (n = 57, 73%) thought that pregnancy tests should be available as part of routine care at public sector clinics, however fewer (n = 52, 67%) felt that this should be a free service. All providers said that there are advantages to promoting pregnancy testing-78% (n = 61) said it would definitely facilitate earlier AnC and abortion presentation and 11% (n = 9) said it would provide an opportunity to educate women about pregnancy and pregnancy prevention. Sixty-five percent (n = 51) said that there were also disadvantages. The main disadvantages mentioned were: staff shortages (n = 23, 29%), a concern that pregnancy testing would promote "irresponsible client behaviour" and result in decreased contraceptive use (n = 34, 43%), and the possibility of client's "abusing" of the pregnancy testing service (n = 23, 29%). Most providers (n = 69, 89%) thought that pregnancy tests should be conducted by community health workers, lay counsellors, clinic assistants, or "anyone available", as opposed to nursing staff. While all clinics had a supply of urine pregnancy tests in stock, only one clinic had a sign announcing that pregnancy tests were available.

## Discussion

This is one of few studies in sub-Saharan Africa to assess the relationship between access to urine pregnancy testing as a means of pregnancy confirmation and gestational age at presentation for pregnancy care [7]. This study has demonstrated that timing of presentation is influenced by access to urine pregnancy testing. Obtaining a urine pregnancy test of one's own accord from a pharmacy is associated with a significant decrease in the gestational age at presentation for both AnC and abortion, independently of all other factors, including age, language, level of education, markers of socio-economic status, employment status, prior pregnancy, and knowledge of other reproductive health services.

While urine pregnancy tests are theoretically available at all public clinics in South Africa, including those in this study, participants who accessed a urine pregnancy test of their own accord did so from a private sector pharmacy. Furthermore, an appreciable number of participants reported that, during their current pregnancy, they had been sent from a public clinic to purchase a pregnancy test at a private pharmacy and then return to the public clinic with the results before care could be initiated. This practice needs examination as women should be able to access urine pregnancy testing free of charge at South African public clinics, as part of routine care; pharmacy urine pregnancy tests may be prohibitively expensive for many women. Also, this study has shown that sending pregnant women who have accessed public services to the private sector for pregnancy confirmation via urine testing contributes to delays in presentation. Some women may not actually return to health care services at all, or in the case of abortion may return too late for a legal procedure. Women sent away from the clinic for confirmation of pregnancy who turn out not to be pregnant will likely not return to the clinic with the negative result. This represents a missed opportunity for contraceptive or pre-conception counselling. Better understanding of provider's reluctance to use urine pregnancy tests in the public sector is needed.

This study has several limitations. The participants had all reached pregnancy care in the public sector and were making a first visit for either AnC or abortion services; there is no data on women who never reached or did return to the public-sector services after being sent way – for example, women seeking abortion who were so delayed that they were denied a legal abortion were not included in this study. Also, this quantitative survey is unable to shed light on complex patient-related factors for late presentation – for this qualitative methodologies will be more informative.

Early AnC presentation is associated with improved health outcomes for mother and child, including decreased maternal mortality/morbidity, decreased risk of pregnancy loss, and decreased neonatal mortality/morbidity [2]. For fully effective AnC, the initial visit should take place as soon as the pregnancy is suspected and certainly before 12 weeks gestation [2]. Similarly, abortions that take place during the first 12 weeks of pregnancy are safer and more cost-effective than second trimester abortions [3,9]. Also, because nurses are allowed to perform first trimester abortions in South Africa, decreasing the numbers of second trimester abortions would make abortion services more accessible and increase the feasibility of abortion becoming a primary level service. Currently, over 30% of legal abortions in South Africa are at a gestational age greater than 12 completed weeks [8].

Clearly, if most women make their first visit for AnC and abortion at 23 and 13 weeks, respectively, pregnancy-related care cannot be optimized for these women. Thus, substantially decreasing the median gestational age at presentation for AnC and abortion care is critical to improving maternal and child health in South Africa, and in other similar settings.

Late presentation for pregnancy care is a well-documented and persistent problem in Sub-Saharan Africa [6]. In this study, the majority of women attending AnC and TOP services had heard of urine pregnancy testing as a means of confirmation of pregnancy. However, few women were aware that a pregnancy could be detected by a urine test one to two weeks after a missed period. An additional concern is that the majority of AnC clients (51%) thought it was appropriate to make the first AnC visit after the first trimester of pregnancy.

This study has shown that obtaining a urine pregnancy test at a pharmacy is associated with an earlier presentation for AnC and abortion services. Greater access to private sector urine pregnancy testing alone, however, is not likely to decrease gestational age at presentation sufficiently, as private sector tests require financial resources and add at least one visit to the care-seeking process. Making urine pregnancy testing routinely available in public clinics and coupling testing with immediate referral to appropriate pregnancy care (or even initiation of care at point of confirming pregnancy) has the potential to significantly decrease gestational age at first visit. The establishment of a pregnancy confirmation clinic, using urine pregnancy testing, at select antenatal clinics in an urban areas of South Africa led to, on average, a 10-week decreased in the gestational age of booking for AnC care [7].

Several specific programmatic recommendations, which may be applicable in diverse healthcare settings, arise from the findings of this study. First, this study identified several client knowledge gaps around pregnancy recognition and care. This misinformation on the part of women requires urgent correction through community- and health service-based education about the importance of early pregnancy recognition and presentation for care. Public awareness campaigns about these issues should be undertaken in general community settings and in all clinics that serve female clients, not only in AnC and abortion services. Furthermore, this study documented a lack of health education material available to address these knowledge gaps. Educational materials, which inform women about the importance of early pregnancy recognition and confirmation, early presentation for care, and of the services available need to be developed.

Secondly, providers need to be armed with clear guidelines and protocols for urine pregnancy testing. These protocols need to clarify the following: that urine pregnancy testing is an on-site, public sector health service, when pregnancy tests should be done and by whom, and the appropriate referrals for and management of clients with positive and negative pregnancy test results.

Thirdly, an appreciable number of providers held negative views about conducting pregnancy testing for teenage clients and about promoting pregnancy testing more generally. These findings indicate the need for values clarification workshops with providers so that they are able to understand the individual, public health, and health care resource-related benefits of early pregnancy confirmation and are, thus, better equipped to provide pregnancy testing services to all clients irrespective of their personal views.

Fourthly, a substantial proportion of women who are ultimately cared for in the public sector confirm their pregnancy in the private sector. Better public-private interaction is critical to ensuring that these women receive optimal and timely care. Public sector program managers need to inform private sector providers of the problem of late pregnancy confirmation and of the appropriate public sector referral routes if test results are positive or negative.

Finally, healthcare providers voiced concerns that if pregnancy testing is promoted, public sector services will be overwhelmed by clients requesting pregnancy tests. And clients cited long clinic waits as a barrier to obtaining pregnancy tests. Therefore, from both the health services and client perspectives, these findings support the need for the establishment and advertisement of 'fast-track' pregnancy confirmation services in public clinics. These services should enable women to bypass the usual clinic wait and quickly obtain a urine pregnancy test conducted by non-professional members of staff (e.g., a community health workers or lay counselors). The testing should be coupled with either immediate referral to appropriate pregnancy care or initiation of care, depending on the range of services available at a particular clinic.

## Conclusion

Given the proven major clinical benefit and public health impact of early presentation for AnC and abortion services, strategies to decrease gestational age at presentation for pregnancy care, such as the increased use of urine pregnancy testing, should be given priority. Urine pregnancy testing is inexpensive, not staff intensive and logistically simple to implement. The implementation of urine pregnancy testing services has the potential to improve both maternal and neonatal health by decreasing gestational age at presentation for AnC and abortion services among women who are pregnant, as well as by channeling non-pregnant women who wish not to become pregnant into contraceptive care.

## Competing interests

The author(s) declare that they have no competing interests.

## Authors' contributions

CM and JM conceptualised and designed the study and oversaw data collection. CM was primarily responsible for data analysis, the interpretation of results and drafting the manuscript. JM assisted in interpretation of results and critically reviewed the manuscript.

## Pre-publication history

The pre-publication history for this paper can be accessed here:


